# Combining Unmanned Aerial Vehicle (UAV)-Based Multispectral Imagery and Ground-Based Hyperspectral Data for Plant Nitrogen Concentration Estimation in Rice

**DOI:** 10.3389/fpls.2018.00936

**Published:** 2018-07-03

**Authors:** Hengbiao Zheng, Tao Cheng, Dong Li, Xia Yao, Yongchao Tian, Weixing Cao, Yan Zhu

**Affiliations:** National Engineering and Technology Center for Information Agriculture, Key Laboratory for Crop System Analysis and Decision Making, Ministry of Agriculture, Jiangsu Key Laboratory for Information Agriculture, Jiangsu Collaborative Innovation Center for Modern Crop Production, Nanjing Agricultural University, Nanjing, China

**Keywords:** UAV, multispectral imagery, ground hyperspectral data, vegetation index, texture index, PNC, rice

## Abstract

Plant nitrogen concentration (PNC) is a critical indicator of N status for crops, and can be used for N nutrition diagnosis and management. This work aims to explore the potential of multispectral imagery from unmanned aerial vehicle (UAV) for PNC estimation and improve the estimation accuracy with hyperspectral data collected in the field with a hyperspectral radiometer. In this study we combined selected vegetation indices (VIs) and texture information to estimate PNC in rice. The VIs were calculated from ground and aerial platforms and the texture information was obtained from UAV-based multispectral imagery. Two consecutive years (2015 & 2016) of experiments were conducted, involving different N rates, planting densities and rice cultivars. Both UAV flights and ground spectral measurements were taken along with destructive samplings at critical growth stages of rice (*Oryza sativa* L.). After UAV imagery preprocessing, both VIs and texture measurements were calculated. Then the optimal normalized difference texture index (NDTI) from UAV imagery was determined for separated stage groups and the entire season. Results demonstrated that aerial VIs performed well only for pre-heading stages (*R*^2^ = 0.52–0.70), and photochemical reflectance index and blue N index from ground (PRI_g_ and BNI_g_) performed consistently well across all growth stages (*R*^2^ = 0.48–0.65 and 0.39–0.68). Most texture measurements were weakly related to PNC, but the optimal NDTIs could explain 61 and 51% variability of PNC for separated stage groups and entire season, respectively. Moreover, stepwise multiple linear regression (SMLR) models combining aerial VIs and NDTIs did not significantly improve the accuracy of PNC estimation, while models composed of BNI_g_ and optimal NDTIs exhibited significant improvement for PNC estimation across all growth stages. Therefore, the integration of ground-based narrow band spectral indices with UAV-based textural information might be a promising technique in crop growth monitoring.

## Introduction

Nitrogen (N) is one of the most important elements for crop growth. In order to ensure high yield, excess N fertilizer was put into the field, which results in severe N leaching and environmental pollution (Ju et al., 2006; Li et al., 2007). Therefore, precision N management is urgent and essential, which might bring significant economic and environmental benefits. Precision N status monitoring is prerequisite for determining optimal N rate. Traditional method for monitoring crop N status was through destructive sampling and chemical analysis, which was tedious and time-consuming. As a nondestructive method, remote sensing techniques have been applied to monitor N status in the past several decades (Filella et al., 1995; Tarpley et al., 2000; Hansen and Schjoerring, 2003; Zhu et al., 2007; Stroppiana et al., 2009; Inoue et al., 2012; Yao et al., 2015; Sun et al., 2017).

Crop N concentration estimation with remote sensing was studied widely (Table 1), and the majority of studies used ground-based hyperspectral reflectance. Vegetation indices (VIs) were commonly used to estimate crop leaf/plant N concentration (LNC/PNC), and new VIs were proposed to improve estimation accuracy (Stroppiana et al., 2009; Tian et al., 2011; Wang et al., 2012). One of the early studies found the red edge and near-infrared ratio performed best in cotton LNC estimation among all the combinations with 20 spectral bands (Tarpley et al., 2000). Matrix plots were commonly used to find the best performing normalized difference vegetation index (NDVI) or ratio vegetation index (RVI) among thousands of wavelength combinations. For example, Zhu et al. (2007) found that the combination of 1,220 and 610 nm as either simple ratio (SR) or a normalized difference index (NDI) performed best in LNC estimation of rice and wheat crops. Tian et al. (2013) reported that SR (R_553_, R_537_) was the optimal combination for rice LNC estimation. Stroppiana et al. (2009) proposed an optimal normalized difference index [NDI_opt_ = (R_553_-R_483_)/(R_553_+R_483_)], which was strongly correlated with rice PNC (*R*^2^ = 0.65), but least correlated with leaf area index (LAI) and aboveground biomass. For PNC estimation in winter wheat, the optimal NDVI or RVI was composed of reflectance in 400 and 370 nm (Li et al., 2010b). Furthermore, Tian et al. (2011) proposed two new three-band spectral indices [R_434_/(R_496_+R_401_) and R_705_/(R_717_+ R_491_)] to estimate rice LNC with hyperspectral reflectance data, and these two indices significantly outperformed other existing VIs in LNC estimation. Similarly, (R_924_-R_703_+2 × R_423_)/(R_924_+R_703_-2 × R_423_) was proposed with hyperspectral data and proved to be significantly related to LNC of both rice and wheat crops (Wang et al., 2012).

**Table 1:** Summary of studies on nitrogen concentration estimation in crops.

**References**	**Species**	**Spectral range**	**Related to**	**Best method**	**Best accuracy (*R*^2^)**
Tarpley et al., 2000	Cotton	350–1,050 nm	LNC	Red-edge and near-infrared ratio	>0.65
Hansen and Schjoerring, 2003	Winter wheat	438–883 nm	LNC	6 principle components	0.71
Stroppiana et al., 2009	Rice	350–2,500 nm	PNC	NDI_opt_: (R_503_-R_483_)/(R_503_+R_483_)	0.65
Li et al., 2010b	Winter wheat	350–1,075 nm	PNC	NDI: (R_410_-R_365_)/(R_410_+R_365_)	0.57
Tian et al., 2011	Rice	350–2,500 nm	LNC	Three-band spectral index: R_434_/(R_496_+R_401_)	0.83
Lebourgeois et al., 2012^*^	Sugarcane	NIR, R, G, B	LNC	SRPI_b_	0.70
Cao et al., 2013	Rice	NIR, RE, G	PNC	REGDVI: red edge green difference vegetation index	0.33
Feng et al., 2014	Winter wheat	350–2,500 nm	LNC	(R_755_+R_680_-2 × R_REPig_)/(R_755_-R_680_)	0.85
Yao et al., 2015	Winter wheat	350–2,500 nm	LNC	SVM with first derivative canopy spectra	0.78
Schirrmann et al., 2016^*^	Winter wheat	R, G, B	PNC	Ratio of the red and green channel	0.68
Liu et al., 2017^*^	Winter wheat	450–950 nm	LNC	Back Propagation (BP) neural network methods	0.97
Van Der Meij et al., 2017^*^	Oat	400–950 nm	PNC	Simple difference (780 nm – 765 nm)	0.68

The references are indexed by the year of publication and summarized with the species examined, the spectral range of the reflectance data, the expression of nitrogen concentration, the analytical method, and the best result within each study. The UAV studies were marked with ^*^.

*NIR, RE, R, G, and B represent near infrared, red edge, red, green and blue bands, respectively*.

From the aforementioned studies, the majority focused on crops LNC and a limited number of studies were on plant N concentration (PNC), which has been taken as an effective indicator of crop N status. When actual PNC is compared to the critical N concentration at the corresponding biomass level, the N nutrition index (NNI) could be obtained for determining crop N nutrition status and guiding N applications for a target yield (Lemaire et al., 2008; Zhao et al., 2016; Ata-Ul-Karim et al., 2017). Therefore, precise PNC estimation is critical and useful for in-season site-specific N management.

Ground-based spectral data has been used to estimate crop PNC, but the estimation accuracy is not so satisfactory (Stroppiana et al., 2009; Li et al., 2010b), especially with a multispectral sensor (Li et al., 2010a; Cao et al., 2013). Because canopy reflectance is dominated by leaves and hardly receive the signal of stem and panicle (after heading stage), and PNC is consisted of leaf, stem and panicle concentration, thus canopy reflectance is hard to explain the variation of PNC. Moreover, ground-based platform is often limited by low spatial coverage and unfavorable weather conditions.

Recently, unmanned aerial vehicles (UAVs) offer particular advantages over other remote sensing platforms with a high spatial resolution, a spectral resolution adapted for a specific purpose (here PNC estimation) and an appropriate revisit time. UAVs have been applied in many aspects related to crop growth monitoring, as summarized in Yang et al. (2017), but few studies about rice N status monitoring could be found. Because canopy structural variable (e.g., LAI and biomass) might greatly influence the interaction between leaves and radiation, which covered the signal of N status, thus making difficult to estimate N concentration (Stroppiana et al., 2009). Furthermore, previous studies have reported that the ultraviolet, violet and blue regions were shown to be consistently important for PNC estimation (Stroppiana et al., 2009; Li et al., 2010b). However, bands from these regions are generally missing from the current UAV-based sensors. Hunt et al. (2005) found crop N nutrition status could not be detectable with UAV RGB imagery. Lebourgeois et al. (2012) used two sensors (RGB and NIR-G-B cameras) mounted on a UAV to detect N status in sugarcane and found the best correlation of LNC with a broadband version of the simple ratio pigment index (SRPI_b_) (*R*^2^ = 0.7) among all indices examined. Furthermore, Schirrmann et al. (2016) found the ratio of the red and green channels from UAV RGB imagery correlated well (*R*^2^ = 0.68) with PNC in winter wheat for only the heading stage. Liu et al. (2017) used UAV imagery to estimate LNC in wheat winter successfully with the cost of hyperspectral camera. Whether rice PNC could be estimated with UAV multispectral imagery at multiple stages remains to be addressed.

Texture is an important characteristic used to identify objects or regions of interest in any images (Haralick et al., 1973), and it has been commonly used for image classification (Laliberte and Rango, 2009; Murray et al., 2010). Since the beginning of twenty-first century, texture from satellite imagery has been used to estimate aboveground biomass but only for the forest (Lu and Batistella, 2005; Sarker and Nichol, 2011; Kelsey and Neff, 2014). UAV imagery takes the advantage of ultra-high spatial resolution, which indicates that texture is also an important source of information (Podest and Saatchi, 2002; Dell'Acqua and Gamba, 2003). However, texture in the UAV imagery was rarely used for crop growth monitoring. In addition, whether combining ground hyperspectral data could compensate for the limited bands of UAV sensors and improve the estimation accuracy of PNC is worthy to be explored. Therefore, the objectives of this study were (i) to explore the capability of UAV-based multispectral imagery in rice PNC estimation with spectral and textural information, and (ii) to improve PNC estimation accuracy through combining ground hyperspectral data and UAV imagery.

## Materials and methods

### Experimental designs

**Figure 1:**
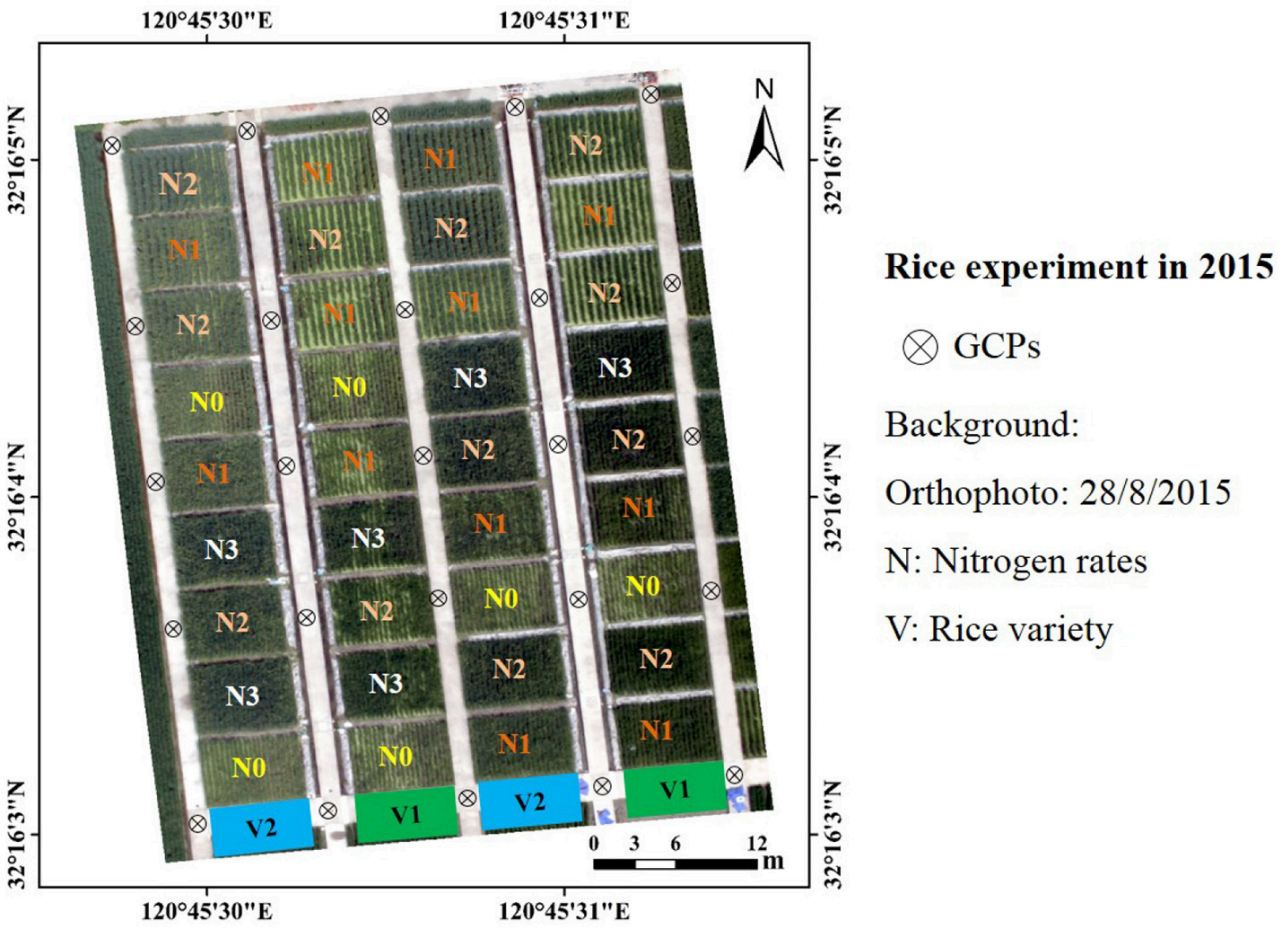
Experimental design: rice experiment at the experimental station of National Engineering and Technology Center for Information Agriculture in 2015; GCPs, ground control points used for band registration and GPS georeferencing.

Two consecutive years' experiments were conducted in the experimental station of National Engineering and Technology Center for Information Agriculture (NETCIA) located in Rugao, Jiangsu province, China (120°45′ E,32°16′ N). The predominant soil type was loam and the organic carbon concentration in the soil was 12.95 g kg^−1^. The annual average temperature, number of precipitation days, and precipitation were about 14.6°C, 121.3, and 1055.5 mm, respectively. In 2015, two rice (*Oryza sativa* L.) cultivars were planted with four levels of nitrogen fertilizer (0 (N0), 100 (N1), 200 (N2) and 300 (N3) kg N ha^−1^ as urea). The treatments with minimum and maximum N rates (N0 and N3) were planted with one density (22 plants m^−2^) and the treatments with intermediate N rates (N1 and N2) were planted with two densities (13 and 22 plants m^−2^). The experiment was organized in 36 plots (5 × 6 m for each plot) with a completely randomized block design (Figure 1).

In 2016, the experiment was similar to the former with same rice varieties. Two rice cultivars were planted with two densities (13 and 22 plants m^−2^) and three levels of nitrogen fertilizer (0 (N0), 150 (N1) and 300 (N2) kg N ha^−1^ as urea). In these two experiments, other field management practices during the experiment followed the local production standards.

### Data collection

#### Ground sampling and N concentration determination

Ground destructive samplings were taken along with the UAV campaigns at rice critical growth stages (Table 2). Three hills of rice plants were randomly selected from the sampling region of each plot and separated into different organs (leaf, stem and panicle). All the samples were oven-dried for 30 min at 105°C and later at 80°C to a constant weight, then weighed, ground and stored in plastic bags for chemical analysis. The total N content in different organs was determined with the micro-Keldjahl method (Bremner and Mulvaney, 1982). The plant N concentration was calculated as:

(1) $$\text{PNC = }(\text{Lw}\times {\text{L}}_{\text{N}}+\text{SW}\times {\text{S}}_{\text{N}}{+\text{P}}_{\text{W}}\times {\text{S}}_{\text{N}})\text{/}({\text{L}}_{\text{W}}{+\text{S}}_{\text{W}}{+\text{P}}_{\text{W}})$$

Where L_W_, S_W_ and P_W_ are the dry weights of leaf, stem and panicle samples, respectively. L_N_, S_N_ and S_N_ are the N concentrations of leaf, stem and panicle samples, respectively.

**Table 2:** Synthesis of experimental design and data acquisition calendar.

**Year**	**Cultivar**	**N rate (kg ha^−1^)**	**UAV flight**	**Spectral measurement**	**Sampling**	**Growth stage**
2015	Wuxiangjing 24 (V1) Yliangyou 1 (V2)	0 (N0), 100 (N1), 200 (N2), 300 (N3)	5 August	28 July	31 July	Jointing
			14 August	14 August	15 August	Booting
			9 September	9 September	10 September	Filling
2016	Wuxiangjing 24 (V1) Yliangyou 1 (V2)	0 (N0), 150 (N1), 300 (N2)	6 August	6 August	6 August	Jointing
			14 August	16 August	14 August	Booting
			28 August	28 August	28 August	Heading
			8 September	9 September	8 September	Filling

#### UAV image acquisition

The UAV used in this study was a multi-rotor Mikrokopter OktoXL (Zhou et al., 2017). It was equipped with a six-band multispectral (MS) camera, a 1.3 Megapixel (1,280 × 1,024) Tetracam mini-MCA6 (Tetracam, Chatsworth, CA, USA) camera with center wavelengths of 490, 550, 680, 720, 800, and 900 nm. The angular field of view is 38.26° × 30.97°, resulting in an individual image footprint of 69 × 55 m, and a nominal resolution of 0.054 m ground sampling distance at 100 m above ground level.

Images were captured at one frame per 3 s and saved as a 10 bit RAW format. Camera settings were adjusted to lighting conditions and set to a fixed exposure for each flight. After the flight, only one image (covering all the 36 plots) was selected for post analysis due to the small study area. All the flights were executed in stable ambient light conditions between 11:00 a.m. and 1:30 p.m.

#### Field spectral measurements

Rice canopy spectral reflectance was collected with an ASD FieldSpec Pro spectrometer (Analytical Spectral Devices, Boulder, CO, USA) with a 25° field of view. The spectral range was 350–2,500 nm, with a 1.4 nm sampling interval between 350 and 1050 nm and a 2 nm sampling interval between 1,000 and 2,500 nm. All the spectral measurements were taken at a height of 1.0 m above the rice canopy from 11:00 a.m. to 1:00 p.m. local time. Three observation points were fixed in each plot and each point was measured five times with the ASD spectrometer. The average of those measurements represented the reflectance spectrum of each plot. Calibration measurements were taken with a white reference panel every 10 min.

#### UAV imagery processing

UAV images were processed in IDL/ENVI environment (Exelis Visual Information Solutions, Boulder, Colorado, USA) and the image preprocessing workflows followed Zhou et al. (2017). Later, band registration was taken with the 25 ground control points (GCPs) to obtain an image with six spectral bands. Radiation correction was conducted with an empirical line correction method (Smith and Milton, 1999; Zhou et al., 2017) by using the six flat calibration canvas at different reflectance intensities (Figure 1). The reflectance of each plot was represented by the average of reflectance values over the non-sampling area of the plot.

#### Calculation of vegetation indices

In this study, canopy spectral reflectance acquired from aerial and ground platforms was used to calculate a number of vegetation indices (Table 3), which were reported to be well correlated with N or chlorophyll concentration. Because multispectral images had only six spectral bands, only NDVI, CI_G_, CI_RE_, OSAVI and VI_opt_ were calculated with UAV imagery.

**Table 3:** Vegetation indices used in this study.

**Vegetation index**	**Equation**	**References**	**Platform**
Normalized difference vegetation index	*NDVI* = (*R*_800_−*R*_680_)/(*R*_800_+*R*_680_)	Rouse et al., 1974	UAV, Ground
Green chlorophyll index	*CI*_*G*_ = (*R*_800_/*R*_550_)−1	Gitelson et al., 2005	UAV, Ground
Red edge chlorophyll index	*CI*_*RE*_ = (*R*_800_/*R*_720_)−1	Gitelson et al., 2005	UAV, Ground
Optimized soil adjusted vegetation index	*OSAVI* = (1+0.16)(*R*_800_−*R*_670_)/(*R*_800_+*R*_670_+0.16)	Rondeaux et al., 1996	UAV, Ground
Optimal vegetation index	$VI_{opt}=\left(1+0.45\right)\left[{\left(R_{800}\right)}^{2}+1\right]/\left(R_{670}+0.45\right)$	Reyniers et al.,, 2006	UAV, Ground
Optimal normalized difference index	*NDI*_*opt*_ = (*R*_503_−*R*_483_)/(*R*_503_+*R*_483_)	Stroppiana et al., 2009	Ground
MERIS terrestrial chlorophyll index	*MTCI* = (*R*_750_−*R*_710_)/(*R*_710_+*R*_680_)	Dash and Curran, 2004	Ground
Photochemical reflectance index	*PRI* = (*R*_570_−*R*_531_)/(*R*_570_+*R*_531_)	Peñuelas et al., 1994	Ground
Blue nitrogen index	*BNI* = *R*_434_/(*R*_496_+*R*_401_)	Tian et al., 2011	Ground

*The bands written correspond to the exact band used in this study. VI from aerial and ground-based platform were distinguished as VI_a_ and VI_g_, respectively*.

#### Texture analysis

Gray level co-occurrence matrix (GLCM) was the most commonly used texture algorithm (Haralick et al., 1973), and employed to test the potential of texture analysis of UAV images on PNC estimation. After the radiation correction was conducted, eight GLCM-based texture measurements [e.g., mean (MEA), variance (VAR), homogeneity (HOM), contrast (CON), dissimilarity (DIS), entropy (ENT), second moment (SEM) and correlation (COR)] were computed with a window size (3 × 3 pixels) in the direction of 45° using the ENVI software. Texture analysis was taken on five bands without 900 nm due to the close correlation between the reflectance of two near infrared bands (data not shown).

Since compared with spectral reflectance data, VIs were shown to reduce the influence of canopy geometry, soil background, illumination angles and atmospheric conditions when estimating biophysical properties (Tucker, 1979; Huete et al., 1985). Therefore, we assumed that texture index with ratio or normalization of texture measurements might have the same function. Then a normalized difference texture index (NDTI = (*T*_1_-*T*_2_)/(*T*_1_+*T*_2_) was proposed, where *T*_1_ and *T*_2_ was random texture measurement from the five bands). In order to select an appropriate texture combination, the correlation between PNC and NDTI was tested by using all possible combinations of texture.

#### Statistical analysis

The data collected from the two-year experiment were pooled to examine the relationships of PNC with VIs, NDTIs and the combinations with simple linear regression (SLR) and stepwise multiple linear regression (SMLR). In order to simplify the estimation model, the number of variables in multiple linear regression (MLR) models was set no more than two. The statistical analysis was executed in Graph-Pad Prism (GraphPad Software Inc., San Diego, CA, USA, 1996) and SPSS 20.0 software (SPSS INC., Chicago, IL, USA, 2002).

The established models were validated with all the data using a k-fold (*k* = 10) cross-validation procedure, and evaluated by the differences in the root mean square error (RMSE) and the relative RMSE (RRMSE). The RMSE and RRMSE were calculated using Equations (2, 3), respectively:

(2) $$\begin{array}{r} RMSE=\sqrt{\frac{1}{n}\overset{n}{\underset{i=1}{\sum }}{\left(P_{i}-O_{i}\right)}^{2}} \end{array}$$

(3) $$\begin{array}{r} RRMSE\left(\%\right)=\frac{100}{\bar{O_{i}}}\sqrt{\frac{1}{n}\overset{n}{\underset{i=1}{\sum }}{\left(P_{i}-O_{i}\right)}^{2}} \end{array}$$

Where $\bar{O_{i}}$, *P*_*i*_ and *O*_*i*_ were the observed, predicted and mean values of rice PNC, respectively, and n was the number of samples.

## Results

### Performance of spectral vegetation indices

Table 4 shows the simple linear relationships between PNC and VIs from two platforms. For aerial VIs, NDVI_a_, and OSAVI_a_ exhibited moderate performance and CI_G−a_ and CI_RE−a_ performed equally well and best amongst all VIs for pre-heading stages. For post-heading stages and the entire season, all aerial VIs were weakly related to PNC with the highest *R*^2^ of 0.28 and 0.14, respectively.

**Table 4:** Simple linear relationship between PNC and vegetation indices (*R*^2^).

**VI**	**Pre-heading**	**Post-heading**	**Entire season**
NDVI_a_	0.52^***^	0.02^ns^	0.02^ns^
CI_G−a_	0.70^***^	0.04^*^	0.02^ns^
CI_RE−a_	0.70^***^	0.28^***^	0.14^***^
OSAVI_a_	0.56^***^	0.28^***^	0.05^**^
VI_opt−a_	0.64^***^	0.28^***^	0.05^**^
NDVI_g_	0.43^***^	0.35^***^	0.10^***^
CI_G−g_	0.61^***^	0.27^***^	0.20^***^
CI_RE−g_	0.63^***^	0.40^***^	0.26^***^
OSAVI_g_	0.48^***^	0.47^***^	0.14^***^
VI_opt−g_	0.54^***^	0.46^***^	0.17^***^
NDI_opt−g_	0.01^ns^	0.00^ns^	0.28^***^
MTCI_g_	0.63^***^	0.35^***^	0.32^***^
PRI_g_	0.51^***^	0.48^***^	0.65^***^
BNI_g_	0.64^***^	0.39^***^	0.68^***^

*VI_a_ and VI_g_ denote VI from aerial and ground-based platform, respectively. The numbers in bold denotes the maximum in each column. Significance level: ns, not significant*,

* *p < 0.05*,

** *p < 0.01*,

*** *p < 0.001*.

*NDVI, Normalized difference vegetation index; CI_G_, Green chlorophyll index; CI_RE_, Red edge chlorophyll index; OSAVI, Optimized soil adjusted vegetation index; VI_opt_, Optimal vegetation index; NDI_opt_, Optimal normalized difference index; MTCI, MERIS terrestrial chlorophyll index; PRI, Photochemical reflectance index; BNI, Blue nitrogen index*.

**Figure 2:**
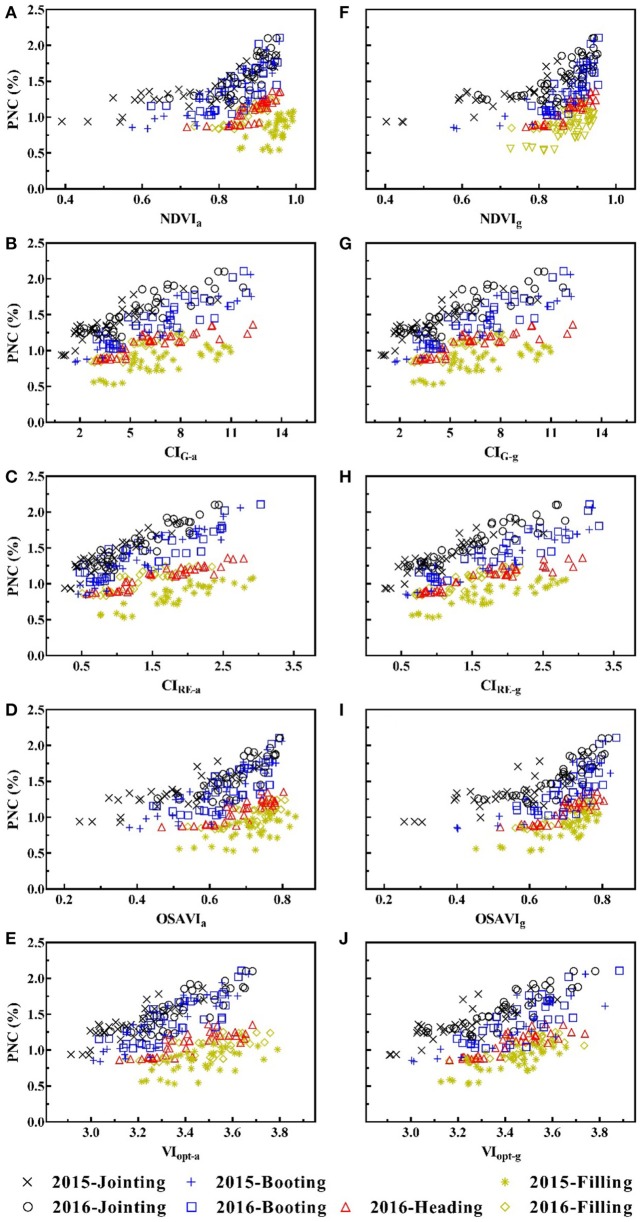
Plant nitrogen concentration (PNC, %) plotted against counterpart vegetation indices from two platforms: **(A)** NDVI_a_; **(B)** CI_RE−a_; **(C)** CI_G−a_; **(D)** OSAVI_a_; **(E)** VI_opt−a_; **(F)** NDVI_g_; **(G)** CI_RE−g_; **(H)** CI_G−g_; **(I)** OSAVI_g_; **(J)** VI_opt−g_. The dashed line is fitted for all data points.

For ground VIs, the majority of VIs only performed well for pre-heading stages. OSAVI_g_ and VI_opt−g_ showed no significant difference in PNC estimation before or after heading stage, while PRI_g_ and BNI_g_ exhibited equal performance cross all growth stages. Compared with VIs from ground-based platform, aerial VIs performed better for pre-heading stages and worse for post-heading stages and entire season (Figure 2).

### Performance of texture features and texture indices

The relationships between PNC and texture measurements of all spectral bands were found to be poor across all growth stages, though stronger correlation was observed with MEA_800_ (*R*^2^ = 0.51), MEA_800_ (*R*^2^ = 0.41) and HOM_720_ (*R*^2^ = 0.42) for pre-heading, post-heading stages and entire season, respectively (Supplementary Table 1). Compared with individual texture measurements, NDTIs performed significantly better in PNC estimation across all growth stages (Table 5). NDTI1, composed by MEA_800_ and MEA_720_, performed best in PNC estimation for the pre-heading stages. For post-heading stages, the top eight best-performing NDTIs were mainly composed by texture measurements from red edge and near infrared bands. The top one was NDTI9 with MEA_800_ and DIS_720_, explaining 61% variability of PNC for the post-heading stages. Similar to the result for post-heading stages, NDTIs showing close relationship with PNC for entire season were all composed of texture measurements in 720 and 800 nm. NDTI17 could explain 50% variability of PNC, which was superior to other NDTIs.

**Table 5:** Simple linear relationship between PNC and the top eight best-performing normalized difference texture indices (*R*^2^).

	**Pre-heading**				**Post-heading**				**Entire season**		
**NDTI**	***T1***	***T2***	***R*^2^**	**NDTI**	***T1***	***T2***	***R*^2^**	**NDTI**	***T1***	***T2***	***R*^2^**
NDTI1	MEA_800_	MEA_720_	0.61	NDTI9	MEA_800_	DIS_720_	0.61	NDTI17	COR_800_	COR_720_	0.50
NDTI2	MEA_680_	MEA_550_	0.50	NDTI10	COR_800_	COR_720_	0.59	NDTI18	MEA_720_	HOM_720_	0.45
NDTI3	MEA_680_	ENT_550_	0.50	NDTI11	MEA_800_	CON_720_	0.56	NDTI19	ENT_800_	DIS_720_	0.42
NDTI4	ENT_720_	MEA_680_	0.49	NDTI12	MEA_800_	ENT_550_	0.53	NDTI20	SEM_800_	HOM_720_	0.42
NDTI5	ENT_800_	MEA_680_	0.48	NDTI13	MEA_800_	ENT_720_	0.52	NDTI21	MEA_800_	CON_720_	0.41
NDTI6	DIS_720_	MEA_680_	0.47	NDTI14	HOM_720_	HOM_550_	0.51	NDTI22	SEM_720_	MEA_720_	0.41
NDTI7	MEA_680_	HOM_490_	0.47	NDTI15	MEA_800_	VAR_720_	0.50	NDTI23	ENT_720_	DIS_720_	0.41
NDTI8	MEA_680_	SEM_490_	0.47	NDTI16	HOM_720_	HOM_490_	0.46	NDTI24	ENT_800_	CON_720_	0.40

*All regressions are statistically significant (p < 0.001). MEA, Mean; VAR, Variance; HOM, Homogeneity; CON, Contrast; DIS, Dissimilarity; ENT, Entropy; SEM, Second Moment; COR, Correlation. The acronyms represent the texture parameter from corresponding band. For example, MEA_800_ represents the mean texture parameter from 800 nm*.

### Performance of VI and NDTI combinations

Table 6 shows the best performance of SMLR models combining VIs and NDTIs. Combining NDTIs and aerial VIs, SMLR models did not show significant improvement in comparison to the optimal VI or NDTI with SLR models across all growth stages. The optimal model for pre-heading stages was still composed of CI_RE−g_ with SLR, while the MLR models for post-heading and entire season were all consisted of the top two best-performing NDTIs.

**Table 6:** Plant nitrogen concentration (PNC) estimates derived using UAV imagery texture indices and spectral vegetation indices from aerial or ground platform with stepwise multiple linear regression.

**Platform**	**Stage**	**Model**	**Optimal PNC estimation model**	***R*^2^**
UAV	Pre-heading	Model-1	PNC = 0.392 × CI_RE−a_+0.93	0.70
	Post-heading	Model-2	PNC = 1.695 × NDTI9 + 0.252 × NDTI10-0.562	0.65
	Entire season	Model-3	PNC = 0.507 × NDTI17 + 2.715 × NDTI18+3.369	0.59
UAV+ground	Pre-heading	Model-4	PNC = 7.066 × BNI_g_ + 0.857 × NDTI1-2.479	0.72
	Post-heading	Model-5	PNC = 4.258 × BNI_g_ + 2.385 × NDTI9-3.144	0.73
	Entire season	Model-6	PNC = 9.286 × BNI_g_ + 0.354 × NDTI17-3.545	0.75

*CI_RE−a_, Red edge chlorophyll index from aerial platform; BNIg, Blue nitrogen index from ground platform. NDTI1 = (MEA_800_-MEA_720_)/(MEA_800_+MEA_720_)*;

*NDTI9 = (MEA_800_-DIS_720_)/(MEA_800_+DIS_720_); NDTI10 = (COR_800_-COR_720_)/(COR_800_+COR_720_)*;

*NDTI17 = (COR_800_-COR_720_)/(COR_800_+COR_720_);NDTI18 = (MEA_720_-HOM_720_)/(MEA_720_+HOM_720_)*.

However, when combining NDTIs and groud-based VIs, the performance of MLR models improved significantly across all growth stages. Interestingly, all the models were consisted of optimal NDTI and BNI, explaining 72, 73, and 75% variability of PNC for pre-headings stages, post-heading stages and entire season, respectively. Therefore, the combination of ground-based VIs and NDTIs with MLR models could be taken as an efficient approach in PNC estimation.

### Model validation

**Figure 3:**
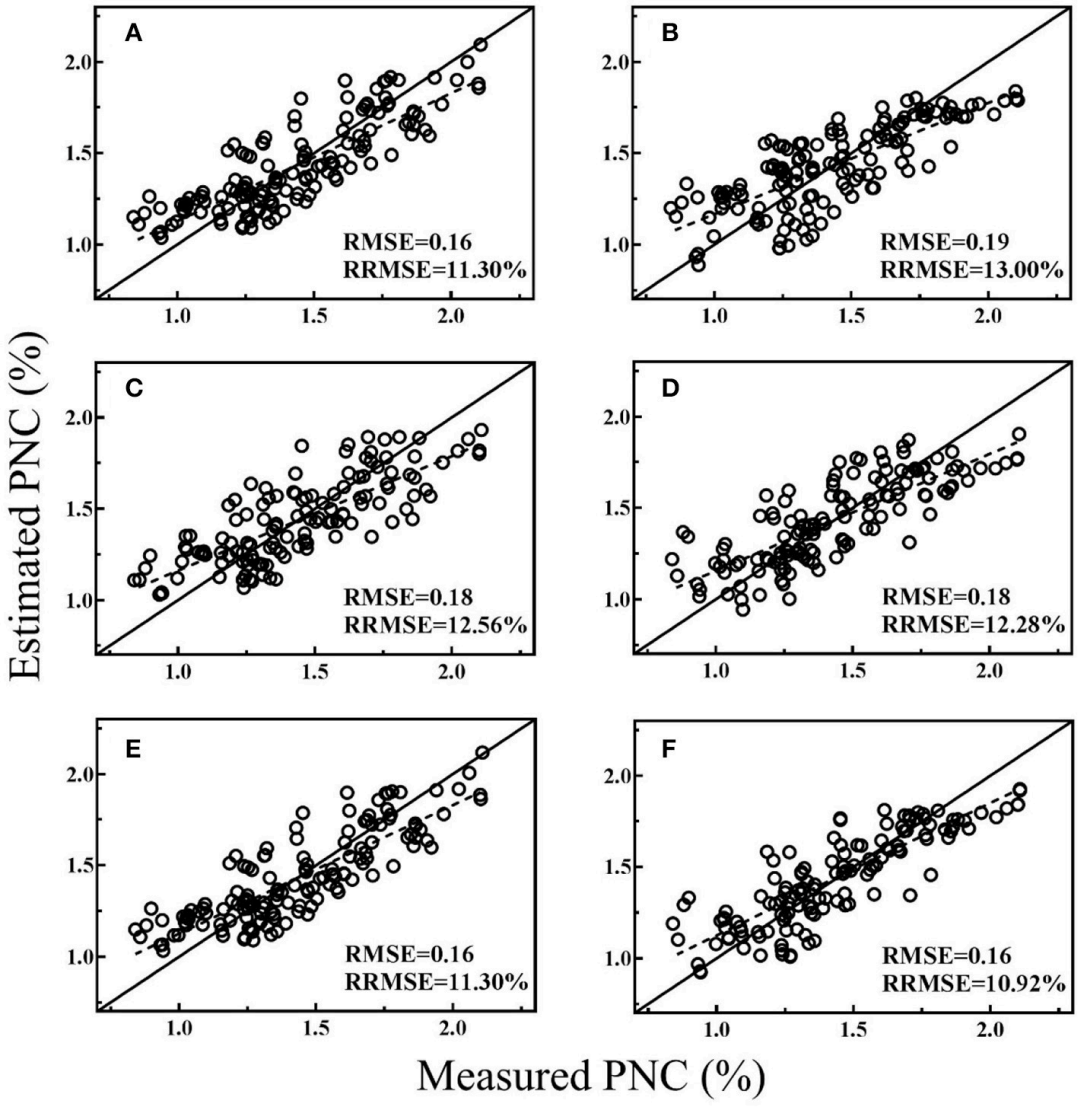
Cross-validation scatter plots for measured PNC vs. estimated PNC derived from selected models for pre-heading stages: CI_RE−a_ **(A)**, NDTI1 **(B)**, MTCI_g_ **(C)**, BNI_g_ **(D)**, Model-1 **(E)**, and Model-4 **(F)**.

**Figure 4:**
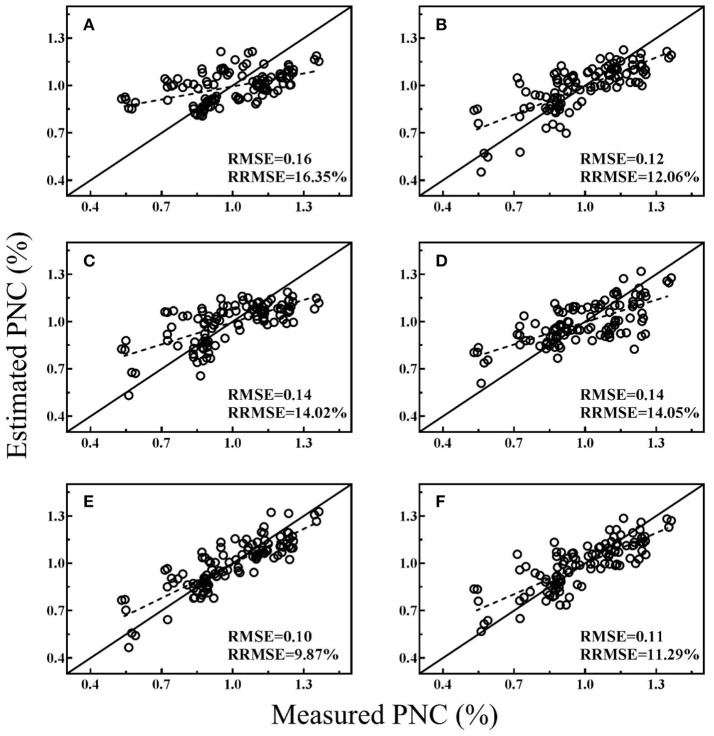
Cross-validation scatter plots for measured PNC vs. estimated PNC derived from selected models for post-heading stages: CI_RE−a_ **(A)**, NDTI9 **(B)**, OSAVI_g_ **(C)**, PRI_g_ **(D)**, Model-2 **(E)**, and Model-5 **(F)**.

**Figure 5:**
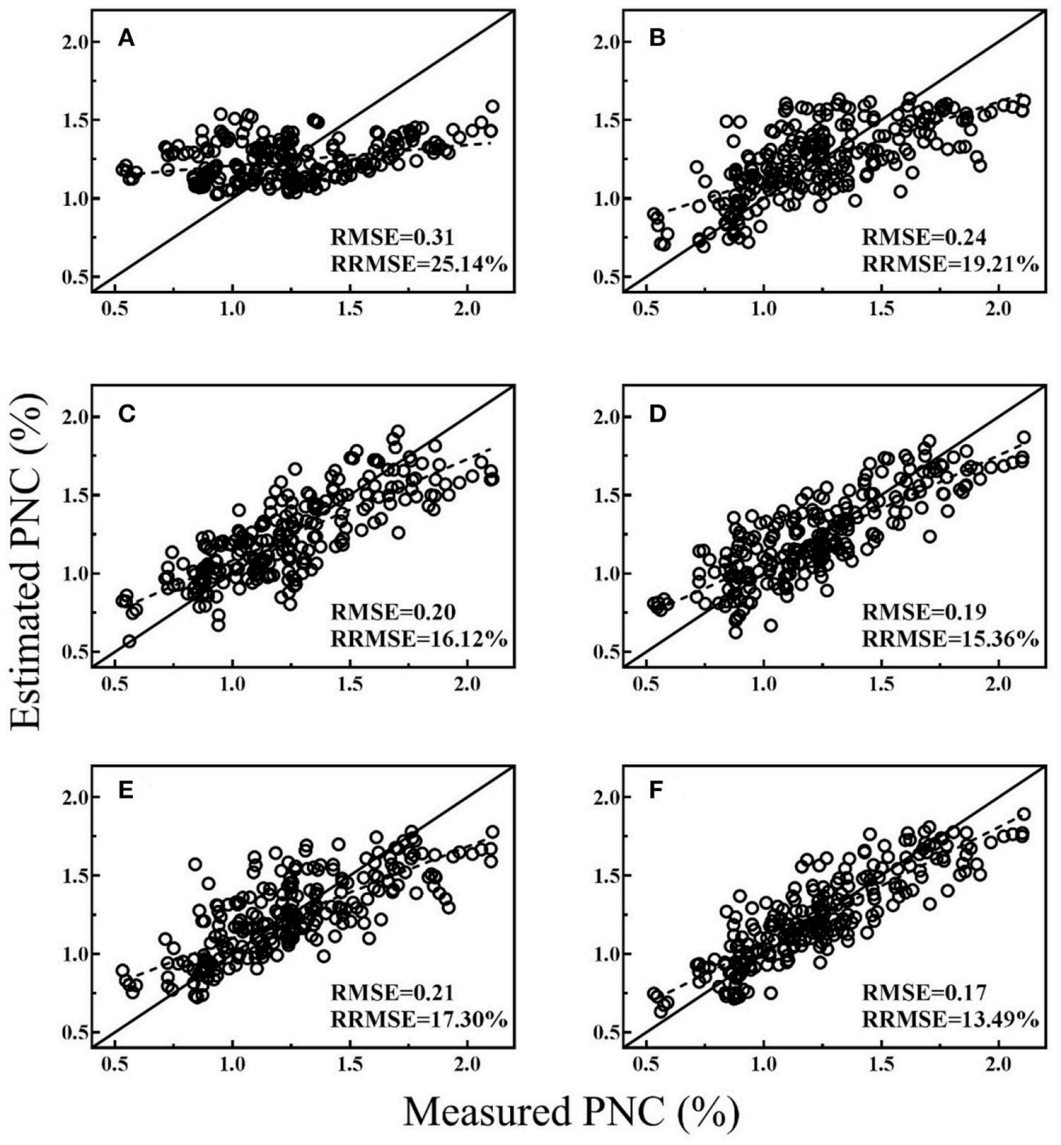
Cross-validation scatter plots for measured PNC vs. estimated PNC derived from selected models for entire season: CI_RE−a_ **(A)**, NDTI17 **(B)**, PRI_g_ **(C)**, BNI_g_ **(D)**, Model-3 **(E)**, and Model-6 **(F)**.

All the regression models were cross-validated with all data and the best performing VI from both platforms, texture index and MLR models were shown in Figures 3–5 for different stage groups. For pre-heading stages, all the selected models had close performance and MLR models showed minor advantages (Figure 3). The highest estimation accuracy (RMSE = 0.16 and RRMSE = 10.92%) was obtained by model-4, composed of NDTI1 and BNI_g_. For post-heading stages, NDTIs exhibited higher estimation accuracy than that of VIs (Figure 4B). Significant improvements were achieved by MLR models, and model-2 produced lowest RMSE and RRMSE (Figure 4E) following with model-5 consisted of NDTI9 and BNI_g_ (Figure 4F). For entire season, PRI_g_ and BNI_g_ performed equally well and were superior to other VIs and NDTIs (Figures 5C,D). However, compared with these two VIs, Model-6 combining BNI_g_ and NDTI17 yielded higher estimation accuracy with RMSE and RRMSE of 0.17 and 13.49%, respectively (Figure 5F).

## Discussion

### Different performance of VIs from two platforms

In this study, counterpart VIs from UAV imagery performed better than that from ground, but only for pre-heading stages (Figure 2). That might be caused by the variation in reflectance extracted from different sampling sizes. For UAV MS imagery, reflectance was extracted from the non-sampling area (around 12 m^2^) within each plot. While the field view of the ASD spectrometer placed at 1 m above the canopy was a circle in a diameter of approximately 0.22 m (around 0.15 m^2^). For post-heading stages, the canopy was more homogenous and the ground-based VIs outperformed the aerial VIs in PNC estimation.

The best-performing VI was CI_RE−a_ before heading stage, which was expected and in agreement with the findings of Li et al. (2010b). At the early growth stages, biophysical parameters (e.g. biomass, LAI) varied greatly and masked the contribution of chlorophyll and N to the canopy reflectance (Haboudane et al., 2002), thus VIs consisted of red edge and NIR bands performed better than other indices (Table 4). However, aerial VIs had weak capability in PNC estimation for post-heading and the entire season, because those VIs, which are sensitive to the canopy structure, became saturated in high biomass level and hard to monitor N status. Furthermore, ground-based VIs performing consistently well in PNC estimation across all growth stages were composed of blue and green bands (Stroppiana et al., 2009; Yu et al., 2013). UAV-based multispectral cameras were equipped with limited bands with broad bandwidth, thus they can not obtain those N concentration specific VIs. Hunt et al. (2005) also found that UAV RGB imagery could not be used to detect crop nutrient status due to the improper bands.

Ground hyperspectral data takes the great advantage of abundant spectral bands and narrow bandwidth, thus it offers more options for VI computation. In this study PRI_g_ and BNI_g_ exhibited good performance across all growth stages, because they were computed with blue and green bands that are specifically sensitive to N concentration, which is consistent with findings from Stroppiana et al. (2009) and Yu et al. (2013). However, the highest correlation between PNC and ground VI was not so satisfactory for post-heading stages with *R*^2^ < 0.50. That might be because the presence of panicles changed the plot structure and affected the spectral signature (Gnyp et al., 2014). In a UAV-based grain yield prediction study, Zhou et al. (2017) also found that the estimation accuracy of grain yield decreased as rice panicles emerged from the sheath at heading stage. Therefore, it is essential to improve PNC estimation for post-heading stages and the entire season with new data source.

### Difference in texture features between stage groups

Texture can be used as a description of spectral feature distribution in spectral image space (Ning, 1998), which might be interpreted with biological meaning as for the spectral features. In this study we found most texture measurements were weakly related to PNC across all growth stages (Supplementary Table 1), which corresponds well to the findings of Lu and Batistella (2005). Besides, Jin et al. (2015) found only MEA texture feature was useful in residue cover estimation in maize. MEA_490_ and MEA_680_ performed well only for pre-heading stages, because reflectance in the visible bands varied slightly at low chlorophyll content levels and saturated at high levels (Hatfield et al., 2008). As a result, the texture features from the visible bands fluctuated slightly and it was difficult to use visible texture features for detecting the variation in PNC. HOM_720_ and MEA_800_ performed well at late growth stages and the majority of texture features at 720 nm were superior to other texture features for the entire season. That might due to the fact that the reflectance at red edge and NIR bands had a broader variation through the growing season and the texture features from these bands could explain more variation in PNC.

However, texture indices performed significantly better than individual texture measurements, which might be similar to advantages of vegetation index that could reduce the influence of canopy geometry and soil background over raw reflectance data (Tucker, 1979; Huete et al., 1985). Sarker and Nichol (2011) also reported that the ratio of texture parameters could improve the estimation accuracy of forest biomass. Given different stage groups, the optimal NDTI was different, because canopy structure varies as rice plants grow, and leaves dominate the canopy before heading stage. After that panicles emerge out from the sheath, which makes the canopy reflectance more complicated due to the difference in leaf and panicle reflectance (Tang et al., 2007). Interestingly, the optimal NDTIs across all growth stages consisted of texture parameters from red edge and NIR bands (Table 5). Since they are good indicators of canopy chlorophyll (Gitelson et al., 2003b, 2005), LAI and biomass (Gitelson et al., 2003a), the NDTIs from those bands performed well in PNC estimation.

Actually, it is still complicated to select an appropriate texture involving window sizes and image bands for a specific research topic. Although numerous studies have reported texture features were useful in biomass (Lu, 2005; Sarker and Nichol, 2011), LAI (Wulder et al., 1998) and residue cover (Jin et al., 2015) estimation, the underlying mechanism of selected texture measurement remains to be better understood. Those questions need to be clarified in the future studies.

### Advantages of combining ground-based spectral data and UAV imagery

The combination of spectral data and texture measurements has been proposed to improve biomass (Lu, 2005; Eckert, 2012), LAI (Wulder et al., 1998) and residue cover (Jin et al., 2015) estimation with satellite data. In present study, we found that the improvement was not pronounced in PNC estimation by combining aerial VIs and NDTIs due to the limited bands of UAV sensors. However, the combination of ground-based VIs and NDTIs improved PNC estimation significantly across all growth stages, especially for the post-heading stages (Table 6). Because ground-based hyperspectral data is available for those VIs that are highly sensitive to N concentration. In addition, texture analysis could efficiently address saturation problems associated with vegetation indices in dense canopies (Kelsey and Neff, 2014) and detect variable canopy structural characteristics well (Eckert, 2012). MLR models integrated both techniques and could explain 75% variability of PNC for entire season with a general model, which was superior to the findings of Li et al. (2010b) and Stroppiana et al. (2009). Therefore, a combination of UAV imagery and ground hyperspectral data could be taken as an effective hybrid method for N status monitoring in rice. Future work will focus on transferring such an integrative methodology presented here to other agronomic parameters estimation.

### Implications for future applications

Most previous studies estimated crop PNC with ground-based hyperspectral data, but the estimation accuracy was moderate (Stroppiana et al., 2009; Li et al., 2010b). Although high accuracy of PNC estimation in rice was obtained by Yu et al. (2013), the optimal estimation model was established by six bands, which was difficult for practical application. In this study we found that CI_RE_ from UAV multispectral imagery could be used to estimate PNC for pre-heading stages. That indicates that UAV imagery might have potential for N diagnose and management based on PNC, before the heading stage (Ding et al., 2003; Cao et al., 2016). Texture information from UAV imagery could be useful for PNC estimation for post-heading stages, which shows that grain yield and quality is predictable with PNC at late growth stages. Therefore, UAV multispectral imagery could be used to estimate rice PNC with independent models for different stage groups.

Furthermore, the hybrid method combining ground-based hyperspectral data and UAV imagery could accurately estimate PNC across all growth stages. As crop growth monitoring techniques develop, multiple sensors from different platforms have been integrated to collect data (Bendig et al., 2015; Tilly et al., 2015). Additionally, UAV-based hyperspectral imaging might execute this method easily. Therefore, this method is feasible and offers technique support for N diagnose and management, and grain yield and quality prediction in the future.

## Conclusions

This work showed UAV-based multispectral imagery could be used to estimate rice PNC with spectral data only for pre-heading stages, but texture information from UAV imagery could be used to estimate PNC across all growth stages with moderate accuracy. PRI and BNI computed with ground-based hyperspectral data performed consistently well across all growth stages. Furthermore, the combination of ground VIs and NDTIs improved the PNC estimation significantly, but the improvement with aerial VIs and NDTIs was not pronounced. Therefore, this hybrid method with ground spectral data and UAV imagery texture information was promising in rice N status monitoring.

Future work should focus on determining optimal texture parameters involving different texture algorithms, window sizes and spectral bands. Moreover, multiple year datasets are needed to evaluate this new hybrid method to improve the robustness and applicability. Most importantly, realizing N diagnose and N management depending on PNC with presented method is more essential and anticipated.

## Author contributions

YZ and TC designed and directed the rice trials at the experimental station of NETCIA in Rugao, China. HZ and DL conducted the field measurements and the collection of samples. HZ processed the images, analyzed the samples and wrote the paper. YZ, TC, XY, and WC gave valuable comments to the manuscript and carried out critical revisions. All authors gave final approval for publication.

### Conflict of interest statement

The authors declare that the research was conducted in the absence of any commercial or financial relationships that could be construed as a potential conflict of interest.
