# Supplementary material for: Combining Unmanned Aerial Vehicle (UAV)-Based Multispectral Imagery and Ground-Based Hyperspectral Data for Plant Nitrogen Concentration Estimation in Rice
Source: Front Plant Sci. 2018 Jul 3;9:936. doi: 10.3389/fpls.2018.00936 (PMC6043795; doi:10.3389/fpls.2018.00936)
Supplement: Supplementary file 1 [file Table_1.DOCX]

Supplementary **Table 1**

Linear relationships between plant nitrogen concentration (PNC) and texture parameters from different bands (R^2^).

|  | 490 nm | | | 550 nm | | | 680 nm | | | 720 nm | | | 800 nm | | |  |
| --- | --- | --- | --- | --- | --- | --- | --- | --- | --- | --- | --- | --- | --- | --- | --- | --- |
| TX | Pre-heading | Post-heading | Entire season | Pre-heading | Post-heading | Entire season | Pre-heading | Post-heading | Entire season | Pre-heading | Post-heading | Entire season | Pre-heading | Post-heading | Entire season |  |
| MEA | 0.38^***^ | 0.02^ns^ | 0.01^ns^ | 0.22^***^ | 0.03^*^ | 0.02^ns^ | 0.39^***^ | 0.02^ns^ | 0.02^ns^ | 0.13^***^ | 0.01 ^ns^ | 0.20^***^ | 0.51^***^ | 0.41^***^ | 0.00^ns^ |  |
| VAR | 0.01^ns^ | 0.06^**^ | 0.00^ns^ | 0.03^*^ | 0.00^ns^ | 0.00^ns^ | 0.06^**^ | 0.04^*^ | 0.00^ns^ | 0.01^ns^ | 0.28^***^ | 0.37^***^ | 0.05^**^ | 0.13^***^ | 0.21^***^ |  |
| HOM | 0.22^***^ | 0.05^**^ | 0.00^ns^ | 0.12^***^ | 0.00^ns^ | 0.01^ns^ | 0.31^***^ | 0.07^**^ | 0.01^ns^ | 0.05^**^ | 0.35^***^ | 0.42^***^ | 0.01^ns^ | 0.28^***^ | 0.13^***^ |  |
| CON | 0.02 ^ns^ | 0.04^*^ | 0.00^ns^ | 0.04^*^ | 0.00^ns^ | 0.00^ns^ | 0.09^**^ | 0.06^**^ | 0.00^ns^ | 0.03^*^ | 0.21^***^ | 0.37^***^ | 0.02^ns^ | 0.18^***^ | 0.20^***^ |  |
| DIS | 0.17^***^ | 0.05^**^ | 0.00 ^ns^ | 0.10^***^ | 0.00^ns^ | 0.01^ns^ | 0.27^***^ | 0.07^**^ | 0.01^ns^ | 0.04^*^ | 0.28^***^ | 0.41^***^ | 0.02^ns^ | 0.23^***^ | 0.18^***^ |  |
| ENT | 0.10^***^ | 0.08^**^ | 0.00^ns^ | 0.07^**^ | 0.00^ns^ | 0.00^ns^ | 0.30^***^ | 0.05^**^ | 0.00^ns^ | 0.03^*^ | 0.18^***^ | 0.37^***^ | 0.11^***^ | 0.05^**^ | 0.11^***^ |  |
| SEM | 0.08^**^ | 0.09^**^ | 0.02^ns^ | 0.07^**^ | 0.00^ns^ | 0.00^ns^ | 0.31^***^ | 0.05^**^ | 0.00^ns^ | 0.04^*^ | 0.18^***^ | 0.35^***^ | 0.11^***^ | 0.06^**^ | 0.10^***^ |  |
| COR | 0.22^***^ | 0.02 ^ns^ | 0.01^ns^ | 0.10^***^ | 0.00^ns^ | 0.00^ns^ | 0.31^***^ | 0.10^***^ | 0.00^ns^ | 0.07^**^ | 0.01^ns^ | 0.18^***^ | 0.30^***^ | 0.33^***^ | 0.07^**^ |  |

Note: The numbers in bold denotes the maximum in each column. Significance level: ns=not significant, * p < 0.05, ** p < 0.01, *** p < 0.001. MEA, Mean; VAR, Variance; HOM, Homogeneity; CON, Contrast; DIS, Dissimilarity; ENT, Entropy; SEM, Second Moment; COR, Correlation.
